# Point-Wise Phase Estimation Method in Fringe Projection Profilometry under Non-Sinusoidal Distortion

**DOI:** 10.3390/s22124478

**Published:** 2022-06-13

**Authors:** Zhuoyi Yin, Cong Liu, Chuang Zhang, Xiaoyuan He, Fujun Yang

**Affiliations:** 1 School of Civil Engineering, Southeast University, Nanjing 211189, China; yzy_seu@seu.edu.cn (Z.Y.); mmhxy@seu.edu.cn (X.H.); 2 School of Science, Nanjing University of Science and Technology, Nanjing 210094, China; liuc@njust.edu.cn (C.L.); zhangc@njust.edu.cn (C.Z.)

**Keywords:** fringe projection profilometry, phase shifting, phase estimation, non-sinusoidal

## Abstract

In fringe projection profilometry, high-order harmonics information of distorted fringe will lead to errors in the phase estimation. In order to solve this problem, a point-wise phase estimation method based on a neural network (PWPE-NN) is proposed in this paper. The complex nonlinear mapping relationship between the gray values and the phase under non-sinusoidal distortion is constructed by using the simple neural network model. It establishes a novel implicit expression for phase solution without complicated measurement operations. Compared with the previous method of combining local image information, it can accurately calculate each phase value by point. The comparison results show that the traditional method is with periodic phase errors, while the proposed method can effectively eliminate phase errors caused by non-sinusoidal phase shifting.

## 1. Introduction

Fringe projection profilometry (FPP) has been widely used for three-dimensional (3D) shape measurements, because of the advantages of high accuracy, large field of view (FOV) and fast measurement speed [1,2,3,4]. It uses predefined spatial intensity patterns for projection, which will be deformed relative to the original pattern due to the undulation of the surface profile. According to the analysis of the deformed intensity pattern, it can be used to retrieve the morphology.

Fourier transform profilometry (FTP) [5] and phase-shifting profilometry (PSP) [2,6,7] are the usual FPP methods. At present, due to the increasing demand for accuracy in modern measurement applications, and as the limitation of measurement speed is being alleviated by the development of hardware, the PSP method is being more widely used. Regardless, the FPP technique requires that the captured fringe images are with sinusoidal gray distribution. However, many factors may affect the fringe quality, such as gamma distortion, defocusing and so on. These make it difficult to calculate the high-precision phase value without nonlinear calibration and correction [8].

At present, the traditional methods mainly solve this problem by calibrating the nonlinear coefficient (gamma), changing the projection process or modifying the projection image. Guo [9] first adopted a method based on histogram statistics to estimate gamma. Ma [10] solved gamma based on Fourier transform through two sets of spatial carrier patterns of different gamma. In the past few years, gamma correction methods based on least squares [11] and the duty cycle of a coded image [12] have been proposed successively. Although many nonlinear calibration methods have been developed and successfully applied, the problem seems to be much more complicated because the camera projector’s nonlinear gamma effect may be changed over time. Other methods are to redesign the projection process. The double three-step method [13] suppresses the error through the internal law. In addition, the double five-step [14], double N-step [15] and three-step with three-frequency [16] methods are also proposed. However, such methods need to project a large number of images, which increases the time cost of a single measurement. The last methods are to optimize the projection pattern so that the image actually collected by the camera has no non-sinusoidal information. Zhang [17] proposed the digital binary defocusing method. However, it has some limitations whereby the accuracy depends on whether the defocusing degree is appropriate. In addition, an end-to-end image optimization method has been proposed [18]. However, this method still has some environmental limitations.

Regardless of the method adopted, it always can be transformed to a complex non-linear solution problem. Recently, the deep learning technique based on neural networks has been developed rapidly. It has shown extensive prospects in various fields. For FPP, it is currently applied mostly to Fourier phase extraction methods [19,20]. However, the precision of the Fourier method is lower than that of the phase-shifting method. There is also an establishment of the relationship between the phase and the height of the object [21,22]. The methods are also applied to phase unwrapping [23,24].

Due to the coincidence of the rapid development time of the FFP technique and the appearance time of a convolutional neural network, the application of a BP neural network in the FFP technique has not been paid enough attention. More importantly, a neural network has not been applied to establish the relationship between phase and gray under non-sinusoidal distortion point-wise. This means that the BP neural network has the potential and feasibility of being applied. Therefore, this paper proposes a point-wise phase estimation method based on a neural network (PWPE-NN). This method solves the issue of the phase errors caused by non-sinusoidal fringe images by establishing an intelligent nonlinear relationship between the pixel gray value and the phase value. It utilizes a flat plane as the calibration object, and achieves accurate phase information in an actual experiment through neural network training.

## 2. Principle

### 2.1. PSP Method

Ideally, under the PSP method, the gray value of *i*-th step *I_i_* at each point can be expressed as:

(1) $$I_{i}\left(x,y\right)=W_{0}(x,y)+W_{1}(x,y)\cos[\theta_{i}+\varphi (x,y)]$$

where (*x*,*y*) represent the pixel position, *W*_0_ is the background light intensity, *W*_1_ is the surface reflectivity, *φ* is the corresponding phase value, and *θ* the shifted phase. *W*_0_, *W*_1_ and *φ* are unknown quantities that need to be solved. A measurement system may contain high-order harmonics, which means

(2) $$\begin{array}{cc} I_{i}^{o}(x,y) & =W_{0}(x,y)+W_{1}(x,y)\cos[\theta_{i}+\varphi (x,y)]+W_{2}(x,y)\cos\{2[\theta_{i}+\varphi (x,y)]\}+\ldots \\ & +W_{k}(x,y)\cos\{k[\theta_{i}+\varphi (x,y)]\}+W_{k+1}(x,y)\cos\{(k+1)[\theta_{i}+\varphi (x,y)]\}+\ldots \end{array}$$

The high-order harmonics in each point of an image are not always the same. At least one parameter *E* is needed to describe the form of higher-order harmonics. *E* contains the internal relationship between higher-order harmonic coefficients. Thus, the function of the pixel point (*x*,*y*) may be expressed as:

(3) $$Sy\left(W_{0},W_{1},\varphi ,E\right)=0$$

Therefore, the function relationship *Sy*_(*x*,*y*)_ contains at least four parameters. In other words, to decouple *φ* in this equation, at least four equations are needed. Therefore, the number of phase shift steps is at least four. To suppress the noise and avoid an odd number of steps, a six-step phase-shifting method is used. The shifted phase of each fringe pattern is 2*π*/6.

The least squares (LS) phase solution method for *N*-step phase shifting can be expressed as:

(4) $$\varphi (x,y)=\arctan2\left[\frac{-\sum_{i=1}^{N}I_{i}(x,y)\sin\frac{2\pi i}{N}}{\sum_{i=1}^{N}I_{i}(x,y)\cos\frac{2\pi i}{N}}\right]$$

This process is simple and appropriate, but the phase value *φ* obtained by the LS method may contain errors caused by higher-order harmonics:

(5) $$\begin{array}{cc} \varphi_{err} & =-\arctan\left\{\frac{\sum_{i=0}^{N-1}\sum_{j=1}^{\infty }W_{j}\cos[j(\theta_{i}+\varphi )]\sin\theta_{i}}{\sum_{i=0}^{N-1}\sum_{j=1}^{\infty }W_{j}\cos[j(\theta_{i}+\varphi )]\cos\theta_{i}}\right\}+\arctan\left[\frac{\sum_{i=0}^{N-1}W_{1}\cos(\theta_{i}+\varphi )\sin\theta_{i}}{\sum_{i=0}^{N-1}W_{1}\cos(\theta_{i}+\varphi )\cos\theta_{i}}\right]\\ & =\arctan\left\{\frac{\sum_{i=0}^{N-1}\sum_{j=2}^{\infty }\left[\left(W_{j+1}-W_{j-1}\right)\sin(j\theta_{i}+j\varphi )\right]}{W_{1}+\sum_{i=0}^{N-1}\sum_{j=2}^{\infty }\left[\left(W_{j+1}+W_{j-1}\right)\cos(j\theta_{i}+j\varphi )\right]}\right\}\\ & \approx k\sin\left[N\cdot \varphi (x)\right] \end{array}$$

where *k* is the estimated error amplitude. It becomes smaller as the number of steps *N* increases. After the phase value of each pixel in the image is solved, the unwrapping operation is carried out. Since the image of the collected dataset is a plane calibration plate, the phase distribution after unwrapping should be approximately in accord with the linear function, because the phase error is regular at a higher frequency [25]. The fitting of the image space can effectively eliminate it. Thus, the precise phase value of each pixel position on [−*π*, *π*] can be obtained. If there is no plane calibration plate with high machining accuracy, high phase-shifting steps can also be used to obtain an accurate phase [25].

### 2.2. Proposed PWPE-NN Method

A general neural network method is divided into three steps: (1) data acquisition or generation (including preprocessing), (2) neural network training and testing (including acceleration under large data and stability under deficient data), and (3) application. In this paper, the generation mode of the dataset is defined first, the appropriate processing mode is adopted for the data, and the input and output mode of the neural network structure are designed. It is also necessary to optimize the fitting neural network for the PSP method. Finally, effective and credible results can be obtained.

#### 2.2.1. Dataset Establishment

Earlier in this paper, many factors have been mentioned that may introduce high-order harmonic information into the captured sinusoidal fringe image. The digital binary image defocusing method is commonly employed because binary images can increase the digital light processing (DLP) projection speed, reduce the fringe period and simultaneously expand the FOV of projection. Therefore, the effectiveness of the proposed PWPE-NN method is verified under different defocusing degrees. However, it is worth mentioning that the application of the PWPE-NN method is not only limited to this scenario.

**Figure 1:**
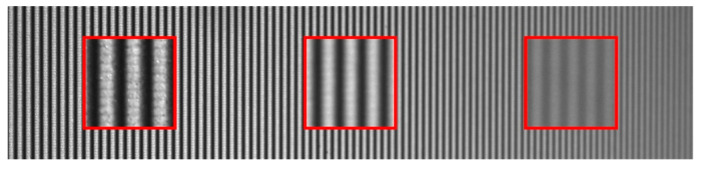
A fringe pattern at different defocusing levels (enlarged in the red box).

In order to simulate the real situation, real experiment images are applied. Figure 1 shows a captured fringe image in the experiments. It can be seen that there is a seam between adjacent fringes (horizontal stripes in vertical fringe); this is because the resolution of the camera is much larger than that of the projector in large FOV measurements. It is difficult to obtain using computer defocusing simulations.

The captured images should include several situations from defocusing to focusing, as shown in Figure 1. Therefore, a plane calibration plate is set up in the measurement system to project a set of phase-shifted binary fringes. The defocusing degree distribution in the image is significantly different through adjusting the angle of the plate. Limited by the depth of field of the camera, the left side of the images is in focus and the right one is out of focus.

The gray values of the same pixel under different phase shifts are selected to form dataset *D*; *i* represents the phase-shifted step, *D* = [*D*(1), …, *D*(*i*), …, *D*(*N*)]. The data vector *D* is normalized to [−1, 1], which can be described as:

(6) $$D_{n}(i)=\frac{2D(i)-\max(D)-\min(D)}{\max(D)-\min(D)}$$

where *D_n_* is the normalized result of *D*, and max and min are the maximum and minimum value of the dataset.

After normalization, the next step is data augmentation. Two optional technical details may be considered.

If the quantity of data is low, the following data expansion techniques can be used:

(7) $$D_{in}\{\begin{array}{c} \left[D(i+1),D(i+2),\cdots ,D(N),D(1),D(2),\cdots ,D(i)\right]\\ \left[D(i),D(i-1),\cdots ,D(1),D(N),\cdots ,D(2+i,)D(1+i)\right] \end{array}$$

where *D_in_* represents the new dataset generated by expansion, *I* = 1, 2…, *N*. Taking the six-step phase-shifting method *N* = 6 as an example, one piece of data can be expanded to 12 pieces of data through data rotation and symmetry.

If the quantity of data is large, the following data acceleration techniques can be adopted.

The largest amount of data in *D* is selected. Assuming that its location is *j*, it can be expressed as:

(8) $$D_{ac}=\left[D(j),D(j+1),\cdots ,D(N),D(1),D(2),\cdots ,D(j-1)\right]$$

where *D_ac_* represents an improved dataset for accelerating the network training. This output value is limited to [−2*π*/*N*, 2*π*/*N*]. Note that because of the existence of random noise, the selection of the maximum value may be difficult; thus, the range of output values is not precise. Once *j* is recorded, its true phase value can be calculated. Experiments show that data augmentation has a positive effect on the stability and accuracy of neural network training.

However, the obtained accurate phase value cannot be directly used as the output value. In a previous work, it was found that if only the phase value is taken as a single output value, a sharp boundary effect will appear near −*π* and *π*. That is to say, there are serious training convergence errors at −*π* and *π*, and the influence of the random noise value on the solution results is particularly severe. This phenomenon is due to the fact that if the phase value is directly taken as the output, there is a distance of 2*π* in the mathematical sense for two values −*π* and *π*, which are equal in the physical sense. That is, there is a mutation that should not exist.

**Figure 2:**
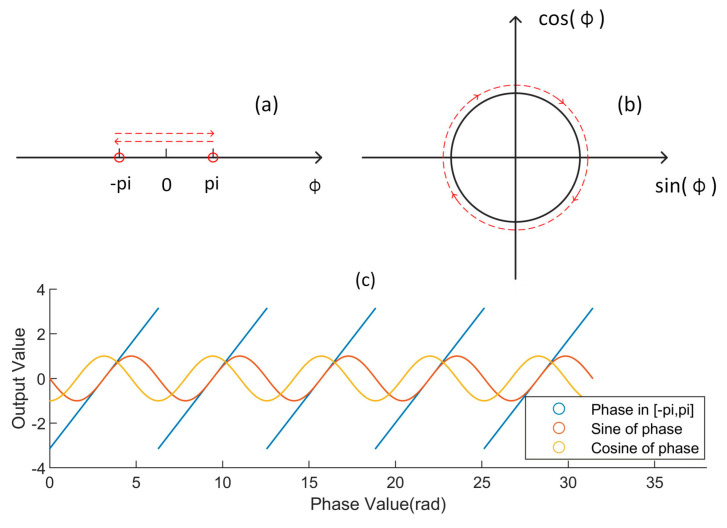
Comparison of the continuity of different output modes. (**a**) Change of phase; (**b**) Change of sine and cosine of phase; (**c**) Comparison of changes.

Therefore, the output value is replaced by the sine and cosine values corresponding to the phase value. As shown in Figure 2, the sine and cosine values are continuously varying, with no abrupt change. This operation is crucial. From later experiments, it is clear that the boundary effect disappears completely. Note that when the sine and cosine values of the phase are known, the phase value can be obtained by an arc tangent operation. This change also has some promising uses. The output values *O_s_* and *O_c_* of the network can be tested to determine whether the solution is correct. The ideal value of $\sqrt{O_{s}^{2}+O_{c}^{2}}$ should be 1. When the difference between $\sqrt{O_{s}^{2}+O_{c}^{2}}$ and 1 increases, there may be some problems in the input data or the trained network. The established solution system forms a self-test.

It can be seen from the previous section that the input data have been expanded, and the corresponding output set only must compensate for several 2*π*/*N*, which are expressed as follows:

(9) $$\varphi_{in}=\{\begin{array}{c} \varphi +i\frac{2\pi }{N}\\ 2\pi -(\varphi +i\frac{2\pi }{N}) \end{array}\begin{array}{cc} & \to \\ & \to \end{array}\begin{array}{c} \left[-\pi ,\pi \right]\\ \left[-\pi ,\pi \right] \end{array}$$

where *φ_in_* represents the compensated output, and → is the value of the phase angle converted to [−*π*, *π*].

#### 2.2.2. Neural Network Training and Testing

This paper uses a feedforward neural network (FNN) model. In addition to the input and output layers, there are three hidden layers, each containing twelve neurons. In order to increase the nonlinear mapping of the system, the activation function *F*(*x*) of each layer of neurons can be expressed as follows:

(10) $$F(x)=\frac{2}{1+e^{-2x}}-1$$

where *x* is the value received by each neuron.

**Figure 3:**
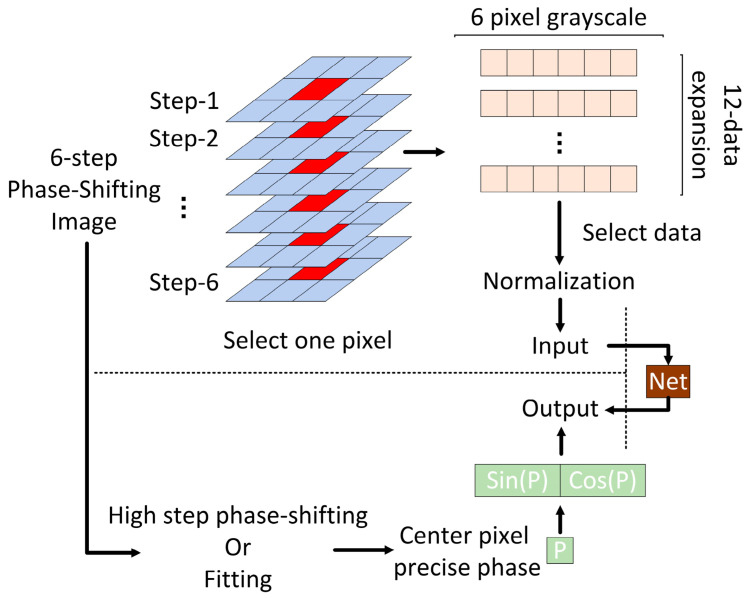
Comparison of the continuity of different output modes.

The flowchart of the PWPE-NN method proposed in this paper is shown in Figure 3, which mainly includes the following three steps: (1) data acquisition and processing, (2) neural network training, and (3) neural network testing.

In this paper, the dataset is collected by the method mentioned above and we randomly scramble them. One part of the dataset is selected as the training dataset (70%), and the other part as the test dataset (30%). GPU (NVIDIA Quadro P3200) and CPU (Intel i7-8850H) muti-threading are used to accelerate the neural network training. After 10,000 iterations, the network converges to the minimum error, and the mean-square error is less than 5 × 10^−4^ rad. In an additional test, only 1% random points are sampled in the test dataset; similar results can be achieved. Thus, the random sampling method can significantly reduce training costs. Only 10 min were required to complete 10,000 training iterations.

## 3. Experiments

**Figure 4:**
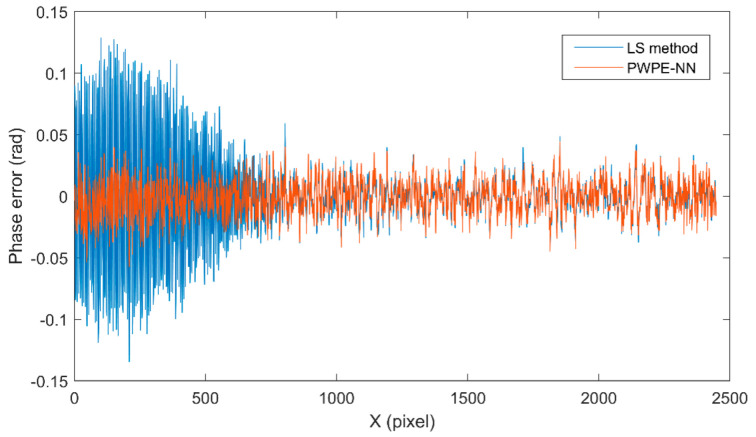
Result of one row in trained image.

In this paper, the plane calibration plate is used as the simulation object. The experimental system uses a Texas DLP6500FLQ projector and a IDS UI-3370CP camera. The resolution of the camera is 2048 × 2048 pixels and the frame rate is 80 fps. The camera is configured with a 35 mm focal length Kowa lens. Firstly, the calibration plate, the camera and the projector are fixed on the optical platform to limit the relative displacement between the parts. In this process, the camera lens direction should be pointed to the plane of the calibration plate, and the calibration plate should be in the middle position in the camera perspective. The projector lens should be aligned to the maximum plane direction of the calibration plate for positioning. Then, the analog dataset is collected and processed. The processed dataset is input into the trained neural network. The phase error is shown in Figure 4. This means that the error keeps decreasing until it is completely affected by random noise. The errors of the PWPE-NN method are basically within ±0.03 rad, while the errors of the LS method basically reach 0.1 rad in the first 500 pixels, and the errors from 500 pixels onwards are basically the same as the method in this paper. This shows that the PWPE-NN method is not only accurate but also more stable.

**Figure 5:**
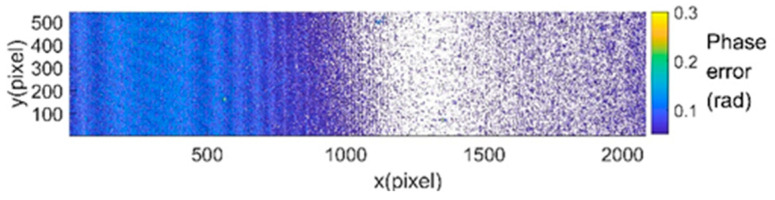
Calculation results of method [8].

Compared with the phase correction method combined with a local image, the proposed method only needs the phase shift data of a single pixel to obtain the phase value. This means that this method has a higher independent resolution. For a measured object with a large gradient or an image with large variation in the high-order harmonics coefficient of a non-sinusoidal wave, the method combined with a local image may face the risk of reducing the accuracy or even failure. Taking method [8] as an example, because it needs to solve an iterative parameter c with the help of the whole image, when the high-order harmonics coefficient of the non-sinusoidal wave in the image changes greatly, the parameter is not constant for the whole measurement image. The image in Figure 1 is taken as an example, and the solution results are shown in Figure 5. This means that the risk of parameter deviation is faced in the process of correction.

**Figure 6:**
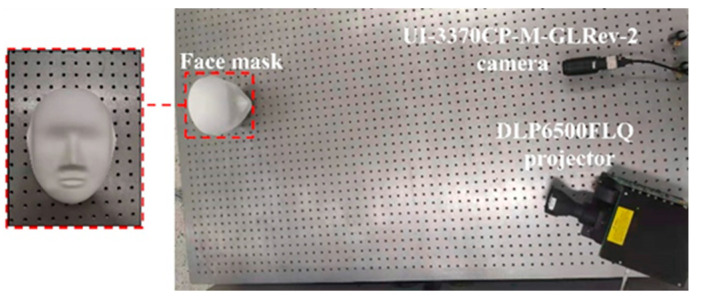
Experimental setup.

To further verify the effectiveness of the proposed method, a face mask is used as the experimental object in Figure 6. The experimental setup is the same as in the previous experiment.

The projector projects six fringe images onto the face mask. The camera is focused directly on a point on the surface of the object. The image corresponding to the six-step phase-shifting method is obtained. A group of phase-shifting images can generate up to 2048 × 2048 × 12 different pieces of data, which is considerable.

**Figure 7:**
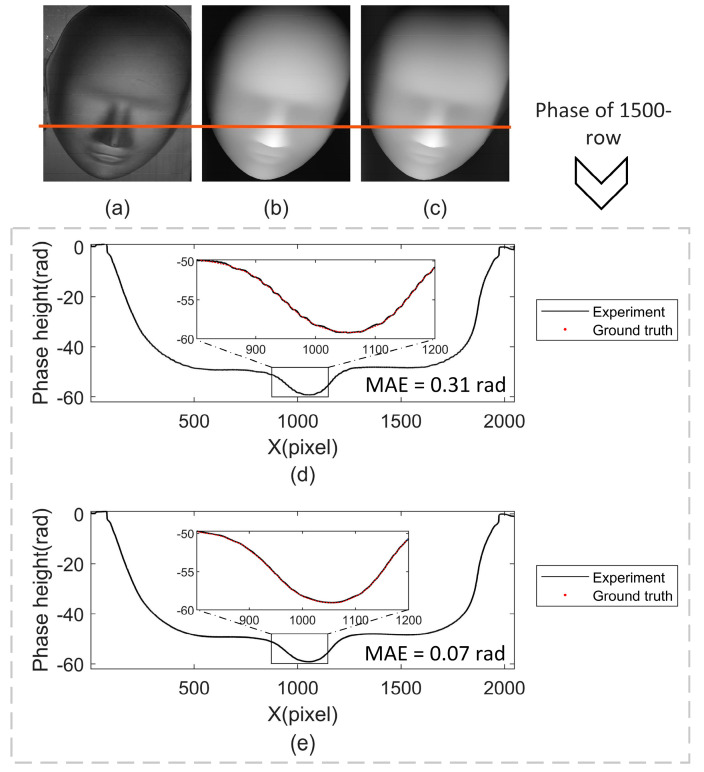
Experimental results of face measurement. (**a**) Object image; (**b**) result of LS method; (**c**) result of PWPE-NN method; (**d**) result of one row with LS method; (**e**) result of one row with PWPE-NN method.

The LS and PWPE-NN methods are used to solve the phase of the face mask. Figure 7a–c, respectively, represent the real image of the face mask, the image obtained by the LS method and the image obtained by the PWPE-NN method. Figure 7d,e show the phase obtained by the two methods at the position of the red line. The phase height near the pixel point 1000 is selected and magnified, and it is obvious that the phase height curve obtained by the LS method fluctuates significantly, indicating that the results obtained by this method have periodic phase errors. However, the phase height curve obtained by the PWPE-NN method is smooth, which indicates that this method can well suppress such errors and has higher precision. In addition, the mean absolute error (MAE) of the phase also illustrates this point.

## 4. Discussion

The application of the method does not depend only on accuracy. On the one hand, the robustness of the method must be confirmed. Whether the accuracy of the method will be greatly reduced due to the influence of noise must be verified. On the other hand, the generalization ability of the method will also determine the difficulty of its application. Easy-to-use methods must remain effective in the face of changing circumstances.

### 4.1. Robustness

**Figure 8:**
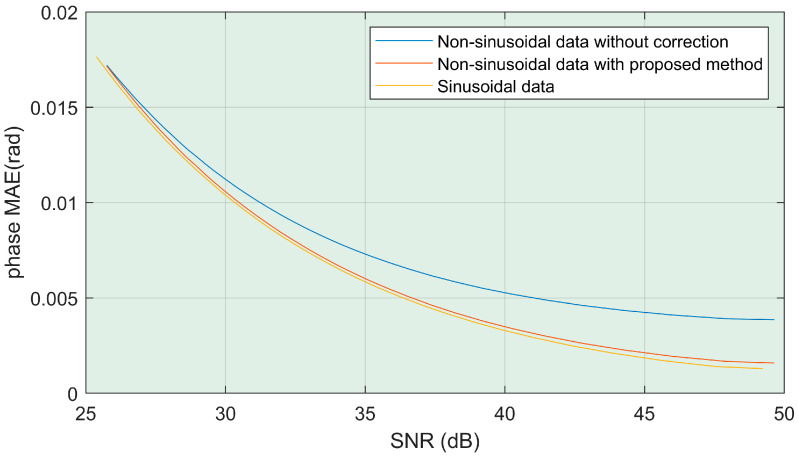
Phase accuracy under different SNRs.

In order to verify the robustness of the method, non-sinusoidal and sinusoidal fringe with different signal-to-noise ratios (SNR) are generated at the same time. The sinusoidal fringe is used as the control. It is used to reflect the influence of noise itself on the error of the phase solution for data without non-sinusoidal distortion. In addition, the LS method and the proposed method are used to solve the non-sinusoidal fringe. The comparison results are shown in Figure 8. It can be clearly observed that the accuracy of the solution in this paper is close to the accuracy under the ideal sinusoidal fringe. Moreover, instability will not occur due to the reduction in the signal-to-noise ratio. This means that this method can solve non-sinusoidal problems well. When the SNR is too low, because the non-sinusoidal information is covered in the noise, the bottleneck of improving the accuracy has shifted to the signal-to-noise ratio. For most industrial cameras, the signal-to-noise ratio is between 40 and 50. This means that our method is effective.

### 4.2. Generalization Ability

The generalization ability of the neural network is examined next. While keeping the relative positions of the projector and camera unchanged, the position of the calibration plate and the focal length of the camera or projector are changed. The first group keeps the position of the calibration plate unchanged and changes the focal length. In groups 2 and 3, the focal length is kept constant and the position of the planar calibration plate in space is adjusted. In group 4, the other settings remain unchanged, reducing the exposure time or aperture.

The LS and PWPE-NN methods are used to calculate the phase of the plane calibration plate. As shown in Table 1, the mean square errors of the two methods in the four groups are obtained. It can be clearly seen that the mean square errors of the PWPE-NN method for solving the four groups of results are smaller, and the stability of the mean square errors is higher. Therefore, compared with the LS method, the PWPE-NN method not only achieves a great improvement in accuracy, but also has relatively stable solution accuracy. At the same time, in order to ensure that the measurement accuracy reaches the limit, the saturation of the light intensity in the image must be ensured. When the spatial position changes, the accuracy will decrease slightly. In the actual measurement, the calibration plane data of multiple positions can be obtained simultaneously for training, which greatly improves the spatial generalization ability of phase calculation.

## 5. Conclusions

The proposed PWPE-NN method can estimate the phase height information of the measured object only by obtaining the gray value of the phase shift image. The proposed method can effectively suppress the phase period errors, and its accuracy is higher than that of traditional methods. This method only needs to be calibrated when the equipment is used for the first time, and there is no need to introduce new steps in the measurement process during subsequent measurements. In addition, we believe that due to its inherent characteristics, the method can be used in many existing phase solution methods. Through integration, this method can help more methods to achieve better results.

The drawback of this method is that it takes more time to solve the phase. On one hand, this can be easily overcome by hardware acceleration, such as through the use of a GPU. On the other hand, in most cases, the importance of accuracy is far greater than calculation time. Moreover, our method still does not eliminate the limitations of datasets. The efficiency of this method will be greatly improved by digital generation instead of the actual collection of available datasets. This is an urgent problem to be solved.

## Figures and Tables

**Table 1:** Mean square error of phase measurement (10^−4^) rad.

	Trained	Group 1	Group 2	Group 3	Group 4
LS	4.626	3.912	5.168	5.233	8.974
PWPE-NN	2.3374	2.4541	2.319	2.961	3.086

## Data Availability

Not applicable.
